# Anaplastic Large Cell Lymphoma of the Spine: Report of a Rare Case

**DOI:** 10.7759/cureus.54602

**Published:** 2024-02-21

**Authors:** Yasir Ali, Ume Hani Naeem, Hefz U Rahman, Sajid Khan, Shafqat Amin, Kamran Ahmad, Haleema Durrani

**Affiliations:** 1 Internal Medicine, Hayatabad Medical Complex Peshawar, Peshawar, PAK; 2 Medical School, Khyber Girls Medical College, Peshawar, PAK

**Keywords:** large cell, atypical presentation, anaplastic, lymphoma, cervical-spine

## Abstract

This abstract discusses a rare case of anaplastic large cell lymphoma (ALCL) involving the cervical and dorsal spine in a 17-year-old female. ALCL is a distinct subtype of lymphoma characterized by abnormal proliferation of lymphocytes and is divided into ALK-positive and ALK-negative subtypes. Spinal involvement in ALCL is uncommon, particularly in the cervical and dorsal regions. The patient presented with persistent fever, weakness, and delayed onset of severe neck pain. Diagnosis involved imaging, bone marrow biopsy, and lymph node biopsy. Treatment strategies for ALCL typically involve a multimodal approach, including chemotherapy, radiotherapy, and targeted therapy. However, due to the rarity of spinal involvement, treatment decisions are based on extrapolation from other ALCL cases. Prognosis is influenced by disease stage and ALK status, but specific outcomes for spinal involvement remain poorly established. This case emphasizes the need for considering lymphoma in patients with unexplained symptoms and abnormal imaging findings. It highlights the importance of further research to improve the understanding and management of ALCL with spinal involvement.

## Introduction

Lymphomas encompass a heterogeneous group of malignancies derived from lymphoid tissue, characterized by the abnormal proliferation of lymphocytes. Anaplastic large cell lymphoma (ALCL) is a distinct subtype of lymphoma, accounting for approximately 2-8% of all cases of non-Hodgkin lymphoma (NHL) [1]. ALCL can occur in both children and adults, with a slight male predominance. It is characterized by the presence of CD30-positive lymphoid cells that exhibit anaplastic morphology, with large, pleomorphic cells and horseshoe-shaped nuclei [2].

The majority of ALCL cases can be classified into two subtypes based on the expression of anaplastic lymphoma kinase (ALK) protein: ALK-positive and ALK-negative. ALK-positive ALCL is commonly seen in children and young adults and is associated with a favorable prognosis [3]. ALK-negative ALCL, on the other hand, occurs more frequently in adults and has a more aggressive clinical course [4].

While ALCL can involve various sites in the body, including lymph nodes, skin, and extranodal sites, its involvement of the spine is relatively uncommon [5]. Among spinal involvements, cervical and dorsal spine involvement is particularly rare [6]. The rarity of spinal involvement in ALCL poses diagnostic challenges and underscores the need for increased awareness among healthcare professionals [7].

ALCL involving the spine presents with a wide range of clinical manifestations, including localized or diffuse back pain, radiculopathy, and neurological deficits, depending on the level and extent of spinal cord compression [8]. The diagnosis of ALCL involving the spine requires a multidisciplinary approach, involving clinical evaluation, imaging studies, histopathological examination, and immunohistochemical staining [9]. Moreover, DUSP22 and TP63 rearrangements predict outcomes in ALK-negative ALCL [10].

## Case presentation

**Figure 1:**
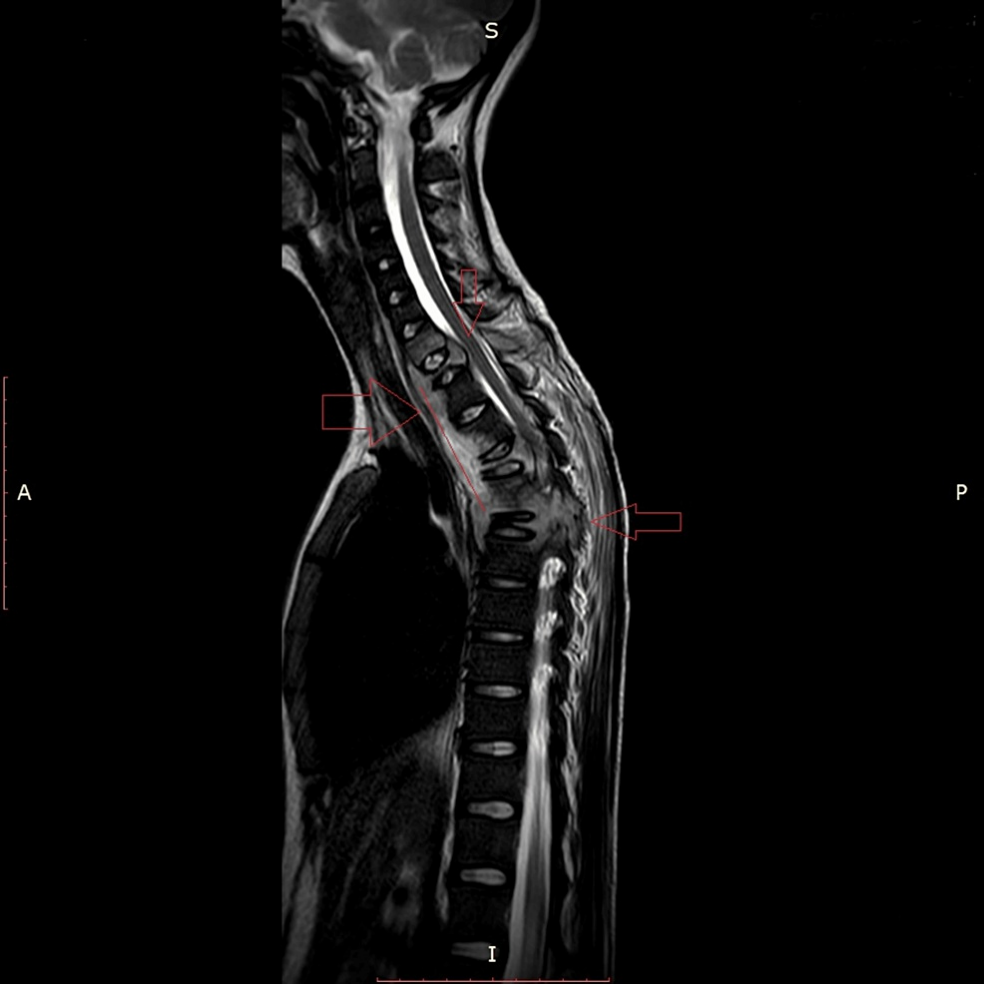
MRI cervical dorsal spine T2W shows altered intensities in the C7, D1, D3, D4, D5, and D6 vertebral bodies and the posterior elements of some of these vertebrae with compression collapse of the D1, D4, and D6 vertebral bodies.

**Figure 2:**
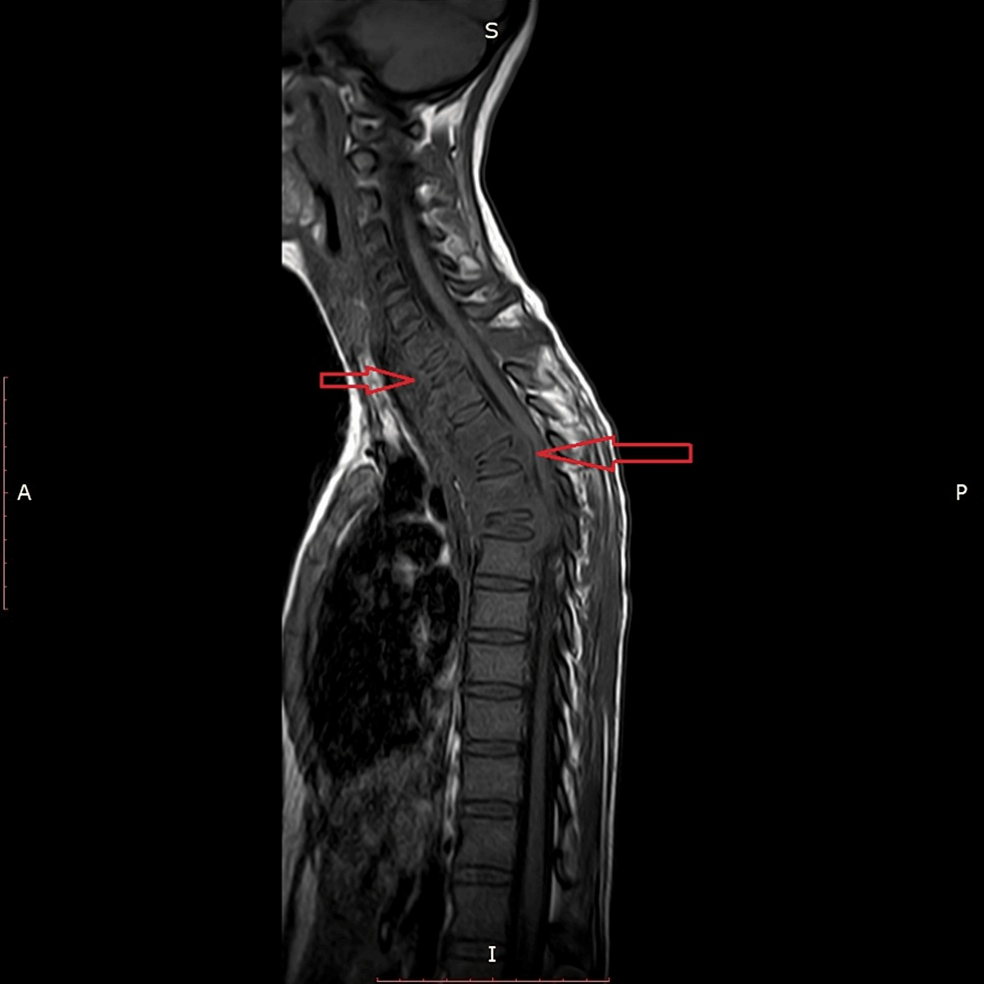
MRI cervical-dorsal spine eT1W showing compression collapse of the D1, D4, AND D6 vertebral bodies. The signal intensities appear hypointense.

**Figure 3:**
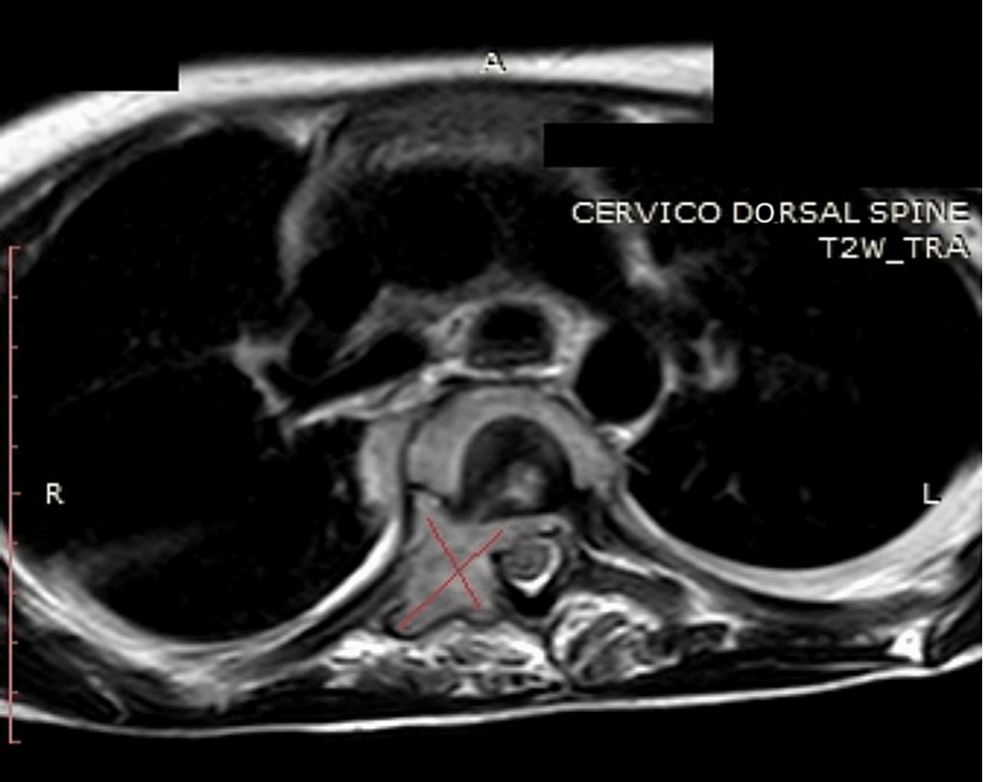
MRI cervical-dorsal spine T2W shows an extension to the paraspinal muscle.

In May 2023, a 17-year-old female presented with a history of persistent fever and generalized body weakness for seven months. Despite extensive workup, including baseline investigations and imaging studies, the cause of her symptoms remained unclear. However, she developed neck pain two weeks prior to her presentation to our ward. The neck pain was severe, exacerbated by neck bending, and accompanied by numbness and paresthesia in the upper limbs. Physical examination revealed inguinal lymphadenopathy. Her baseline investigations were unremarkable. Her peripheral smear revealed hypochromic, microcytosis, and rouleaux formation. Subsequently, her bone marrow biopsy was carried out, which showed a hypercellular marrow with anemia of chronic disease. Further imaging studies, including a CT scan of the spine, demonstrated marked soft tissue thickening in the prevertebral and paravertebral region of C7-D6, extending into the spinal canal and causing erosions and destruction of the adjacent structures. Subsequent MRI of the whole spine revealed altered signal intensity from C7 to D6, with compression collapse of the vertebral bodies and epidural enhancing components causing compression of the spinal cord as shown in Figures 1-3.

**Figure 4:**
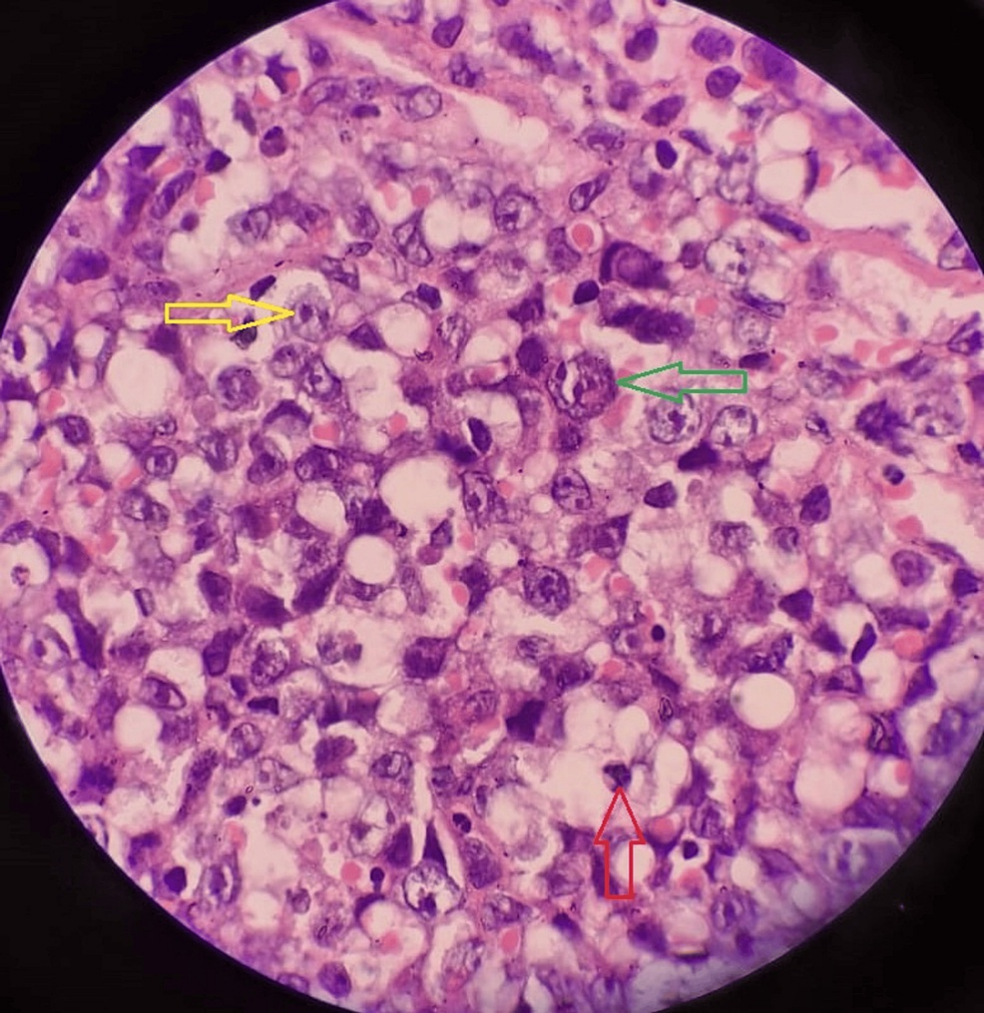
Inguinal lymph node biopsy The yellow arrow points to prominent nucleoli, the green arrow points to mitosis and pleomorphism, and the red arrow also points to the pyknotic nucleus.

Considering these findings and the suspicion of malignancy, an inguinal lymph node biopsy was performed, confirming the diagnosis of anaplastic large-cell lymphoma as shown in Figure 4. Immunohistochemical stains were positive for LCA, CD30, CD3, and Ki-67, further supporting the diagnosis. The patient was subsequently referred to the oncology department for emergency radiation therapy.

## Discussion

The diagnosis of ALCL involving the spine is challenging due to its rarity and the overlap of clinical and radiological features with other neoplastic and non-neoplastic conditions [11]. The persistent fever, generalized weakness, and delayed onset of severe neck pain further complicate the diagnostic process [12]. In our case, the diagnosis was established through a combination of imaging findings, bone marrow biopsy, and lymph node biopsy.

The treatment approach for ALCL typically involves a multimodal approach, including chemotherapy, radiotherapy, and targeted therapy [13]. However, there is limited evidence regarding the optimal treatment strategy specifically for ALCL involving the spine, primarily due to its rarity. Treatment decisions are typically based on extrapolation from the management of ALCL in other sites.

In cases of ALCL with spinal involvement, the primary goal of treatment is to achieve a cure if possible and it is also important to control the disease, alleviate symptoms, and preserve neurological function [9]. The choice of treatment modalities depends on factors such as disease extent, patient age, performance status, and ALK status. Chemotherapy regimens incorporating anthracyclines, such as CHOP (cyclophosphamide, doxorubicin, vincristine, and prednisone) or CHOEP (CHOP plus etoposide), are commonly used [14]. Radiation therapy may be employed as a consolidative treatment to target localized disease or as palliative therapy to alleviate symptoms [15].

ALK-positive ALCL has shown favorable responses to targeted therapy with ALK inhibitors, such as crizotinib or ceritinib, particularly in cases with systemic disease or relapse. The role of targeted therapy in ALK-negative ALCL is still being investigated, and further research is needed to determine its efficacy.

The prognosis of ALCL involving the spine is influenced by various factors, including disease stage, ALK status, and response to treatment. ALK-positive ALCL generally has a more favorable prognosis compared to ALK-negative ALCL [2,3]. However, due to the rarity of this specific manifestation, prognostic factors and survival outcomes specific to spinal involvement are not well established.

## Conclusions

In conclusion, we present a rare case of anaplastic large cell lymphoma involving the cervical and dorsal spine in a 17-year-old female. The diagnosis was confirmed through histopathological examination and immunohistochemical staining. Given the rarity of spinal involvement in ALCL, this case underscores the importance of considering lymphoma as a differential diagnosis in patients presenting with unexplained symptoms. Further studies are needed to better understand the clinical characteristics, optimal diagnostic approaches, and treatment strategies for this unique presentation of ALCL.
